# Does Dietary Supplement Use Increase Micronutrient Intake Adequacy in Healthy Adults with Habitual Omnivorous, Vegetarian, Vegan, and Low-Carbohydrate High-Fat Diets?

**DOI:** 10.3390/nu16121832

**Published:** 2024-06-11

**Authors:** Nives Bogataj Jontez, Karin Šik Novak, Zala Jenko Pražnikar, Ana Petelin, Saša Kenig, Nina Mohorko

**Affiliations:** University of Primorska, Faculty of Health Sciences, 6310 Izola, Slovenia; 97200376@student.upr.si (N.B.J.); karin.novak@fvz.upr.si (K.Š.N.); zala.praznikar@fvz.upr.si (Z.J.P.); ana.petelin@fvz.upr.si (A.P.); sasa.kenig@fvz.upr.si (S.K.)

**Keywords:** vitamin D, vitamin B_12_, potassium, calcium, multimicronutrient dietary supplement

## Abstract

Diets omitting whole food groups pose a risk for micronutrient insufficiencies, but there are no data as to whether those are suitably attenuated with dietary supplements (DS). Micronutrient intakes with food and DSs were analyzed in 130 healthy adults: 32 vegans, 37 vegetarians, 24 following low-carbohydrate high-fat diet (LCHF), and 37 omnivores. A total of 63% used DS (84% of vegans, 75% of LCHF, 54% of vegetarians, and 46% of omnivores); however, a DS did not always tackle dietary insufficiencies. Vitamin B_12_ was often supplemented in vegans in doses substantially higher than recommended, but it was supplemented less often in vegetarians, despite the low prevalence of sufficient intake. Only 43% of participants supplemented vitamin D in wintertime, 23% of them with an insufficient dose. Supplementation of potassium, calcium, and iodine was rare, despite low intake adequacy with food alone in all groups. Some micronutrients were supplemented unnecessarily, such as vitamin K, riboflavin, biotin, and iron. Multimicronutrient DSs were used often; they increased intake adequacy of group B vitamins but failed to sufficiently supplement vitamin D, potassium, calcium, and iodine. Although DS use increased micronutrient intake sufficiency when used properly, the knowledge on micronutrient inadequacy in all dietary patterns should be increased and the public should be educated on the proper use of DSs. Multimicronutrient DSs should be reformulated to tackle the insufficiencies.

## 1. Introduction

The popularity of nutritional patterns excluding whole food groups such as vegetarian, vegan, and low-carbohydrate high-fat (LCHF) diets are rising in areas where the traditional diet has been omnivorous [1], such as Europe, including Slovenia [2]. The vegetarian diet excludes meat, meat products, and often fish, while the vegan diet excludes all foods from animal origin such as milk and milk products, eggs, as well as animal products like honey. The LCHF diet excludes all starchy foods and foods containing sugar. Micronutrients are present in different amounts in different foods and food groups and omitting a whole food group raises the risk of low intake of micronutrients that are abundant in the omitted food group, and consequent micronutrient deficiency [3]. Adequate micronutrient intake is crucial to avoid diseases caused by malnutrition, such as anemia, osteoporosis, rickets, and others [4]. Furthermore, micronutrient adequacy is also important for optimal immune system function [5] and for preventing chronic noncommunicable diseases, such as thyroid deficiency, cardiovascular diseases, and cancer [6]. The literature identifies different micronutrient insufficiencies in different dietary patterns [7,8]. Very few data are available on the habitual LCHF diet practiced without supervision. The most commonly reported deficiency in the vegan diet is vitamin B_12_ deficiency, which can occur also in vegetarians, as vitamin B_12_ is present only in foods from animal origin [9]. Vitamin D intake is generally very low across the population, with especially low intakes in vegans and vegetarians [7,10]. Additionally, in Slovenia, there is a high prevalence of vitamin D deficiency, especially in the winter months when the endogenous production of vitamin D is insufficient due to the angle of the sun [11]. In those practicing LCHF diets, low vitamin C intake was reported [8]. The highest vitamin C intake was previously shown in the vegan group, followed by vegetarians and was lowest in the omnivorous group, but the study did not have a LCHF group [7]. The highest intake of folate was reported in vegans, followed by vegetarians and omnivores [7]. Slovenian national data showed low intakes of folate in 58% of the adult population (mostly omnivores) and folate deficiency in 7.6% of adults [12]. A Swedish study on real-life LCHF diet showed sufficient folate intake in the LCHF group [8]. Low calcium intake was observed in vegans [7,10] and the LCHF diet [13]. Magnesium intake was the highest in the vegan group [7], but usually vegan diets also contain the highest amounts of phytic acid, which inhibits its absorption. The LCHF diet was shown to have low magnesium intake [8,14], and low intakes of iron and potassium were also reported [8].

Nutritional deficiencies can be corrected with dietary supplements (DS), concentrated forms of nutrients or other substances with physiological value [15]. DS use is rising worldwide [16,17,18] and it is assessed that about half to two-thirds of people in Western countries take at least one DS [18,19]. In the USA, multimicronutrient DS (MMN DS) are the most common among DS users, followed by vitamin C [19]. DS users are more likely to adopt positive health-related habits and are often interested in nutrition and its health benefits [19]. It was shown that DS use increases with age, income, and education, it is also known that women are more likely to take DSs [17,19]. People with higher diet quality are more likely DS users [17], but little is known about DS use in different dietary groups such as vegans, vegetarians, and people practicing the LCHF diet.

The aim of this study was to compare micronutrient intake adequacy and DS intake in three habitual nutritional patterns excluding whole food groups and the omnivorous (traditional) dietary pattern in healthy lean adults with matching body mass index (BMI).

## 2. Materials and Methods

### 2.1. Study Design and Participants

A total of 130 participants (97 females), highly interested in healthy nutrition, that self-classified in four dietary groups (omnivorous, vegan, vegetarian, and LCHF) participated in this cross-sectional study. They were included based on the following inclusion criteria: BMI between 18.5 and 30 kg/m^2^, 20–60 years old, and at least six-months adherence to one of the four eligible dietary patterns. Exclusion criteria included change in body mass (more than 3 kg in the preceding 3 months), any chronic disease, taking medications (except contraception) and being pregnant or lactating. All participants were healthy adults, without any chronic diseases.

This study was approved by the Slovenian National Medical Ethics Committee (No. 0120–557/2017/4) and registered at ClinicalTrials.gov, accessed on 8 August 2023. (NCT04347213). Recruitment and protocol have been described in detail previously [2].

### 2.2. Dietary Assessment

Diet was recorded in detail with three-day food diary (two weekdays and one weekend day) and validated food frequency questionnaire (FFQ, [20]). The participants weighed and recorded all foods and beverages immediately before eating and weighed all leftovers. Labels and recipes for mixed dishes were included. Dietitian checked all three-day food diaries and FFQs and any vagueness or questions were clarified on the day the participants came to the Faculty of Health Sciences for measurements in the same week. Three-day food diaries and FFQ were analyzed with the Open Platform for Clinical Nutrition (OPEN, http://opkp.si/, accessed on 20 March 2024). OPEN includes data on micronutrient content from the Slovenian food composition database [21], Souci Fachmann Kraut database [22], and United States National Nutrient Database for Standard Reference [23]. Analysis returned energy intake and energy density, macronutrient intake, and micronutrient intake.

All DSs were recorded in detail, the dose taken was provided. DS data were manually analyzed from the labels and data provided by the manufacturers. For the purpose of this study, the term multimicronutrient DS (MMN DS) will be used for DSs containing three or more vitamins and/or minerals. Number of DS taken, and dose and frequency of intake were determined for all participants.

For the report of each micronutrient intake, the participants were further subdivided within the groups into DS users and nonusers. Intakes of each micronutrient with food alone and with food plus DS are reported in Results Section 3.3. 

Prevalence of adequate micronutrient intake was calculated for each dietary group and further for nonusers and DS users, with food alone and with added DS. Slovenian recommendations for micronutrient intake were used as reference values [24].

Healthy eating index (HEI) was calculated from three-day food diaries for diet quality assessment, as described before [2].

### 2.3. Lifestyle Assessment

Body mass was measured, after an overnight fast of at least 12 h, in light clothing and without shoes with bioelectric impedance analyzer Tanita BC 418MA (Tanita Corporation, Arlington Heights, IL, USA). Height was measured and BMI was calculated.

Participants completed Lifestyle Questionnaire, International Physical Activity Questionnaire (IPAQ), and Socio-Economic Questionnaire, as described before [2].

### 2.4. Statistical Analysis

Statistical analysis was performed using IBM SPSS Statistics 26.0 (IBM, Armonk, NY, USA). Means and standard deviations were calculated. The prevalence of participants with adequate intakes was calculated. The normality of data distribution was evaluated with the Shapiro–Wilk test. On the basis of the data normality distribution, ANOVA and the Kruskal–Wallis’ test were used to compare groups, and Student’s *t*-test and the Mann–Whitney test were used to compare two groups. Pearson’s and Spearman’s correlation were used to evaluate associations between parameters. *p*-values < 0.05 were considered statistically significant.

## 3. Results

### 3.1. Participants’ Characteristics

The participants were divided into four groups based on nutritional pattern: omnivorous, vegan, vegetarian, and LCHF groups (Table 1). The groups were comparable in BMI and energy intake, while macronutrient distribution significantly differed among groups [2] (Table 1). The vegan group reached the highest proportion of recommended intake of carbohydrates, followed by vegetarians and omnivores (84.4%, 54.1%, and 21.6%, respectively). Fat intake below the highest recommended value (RV) was also seen in the highest prevalence in vegans (53.1%), followed by vegetarians and omnivores (21.6% and 18.9%, respectively). On the contrary, all participants in the LCHF group reached the recommended protein intake, while 8.1% of omnivores, 31.2% of vegans, and 35.1% of vegetarians did not reach the RV for protein. The highest intake of dietary fibers was seen in vegans, where 71.9% reached the RV, followed by vegetarians, omnivores, and the LCHF group, where 43.2%, 35.1%, and 8.3% reached the RV [2].

### 3.2. Dietary Supplement Use

**Figure 1:**
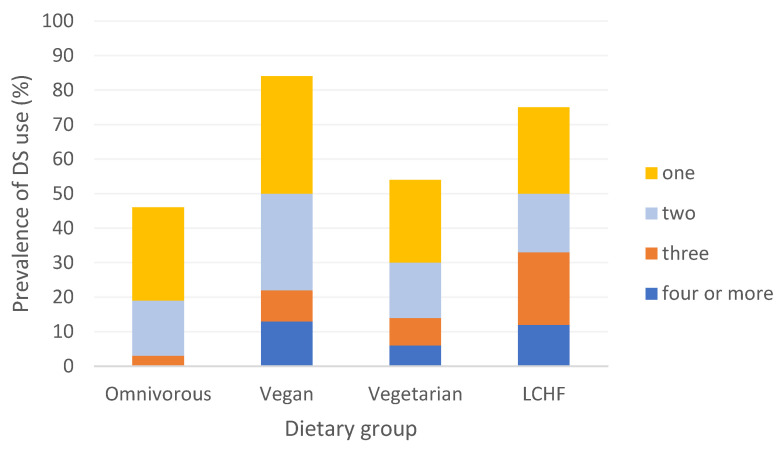
Prevalence (%) of micronutrient dietary supplement use and number of DS used at the same time by participants in a dietary group. DS—dietary supplement; LCHF—low-carbohydrate high-fat.

Almost two-thirds of participants were taking at least one DS. The four most frequently used DSs were vitamin B_12_, vitamin D, vitamin C, and MMN DS. The highest prevalence of DS use (Figure 1) was in the vegan group (84.4%), followed by the LCHF (75.0%), vegetarian (54.1%), and omnivorous group (45.9%), with a significant difference between groups (*p* = 0.004). One DS was taken by 27.7% of participants, two by 19.2%, three by 9.2% and four or more DSs by 7.0% of participants. The highest prevalence of four or more DSs being used at the same time was reported in the vegan and LCHF group, while no participants from the omnivorous group took four or more DSs (Figure 1). The LCHF group also had the highest prevalence of use of three DSs. The choice of micronutrients that were supplemented differed among the groups (Table 2).

In addition to DSs including one or more micronutrients, 36.9% of participants were taking other DSs. The most common ‘other’ supplements were probiotics, maca (*Lepidium meyenii*), methylsulfonylmethane (MSM), collagen, immunobiotics, and creatine. We will only consider micronutrient DSs in this article.

The prevalence of participants taking DSs did not differ between genders (M 51.5%, F 67.0%; *p* = 0.141). There was no significant difference between DS users and nonusers in age (Z = −0.490; *p* = 0.624), physical activity assessed with IPAQ (Z = −0.440; *p* = 0.660), smoking prevalence (Z = −0.309; *p* = 0.757), alcohol consumption (Z = −0.963; *p* = 0.334), education (Z = −0.986; *p* = 0.324), fruit intake (Z = −0.014; *p* = 0.988), vegetable intake (Z = −1.112; *p* = 0.266), diet quality determined with HEI (Z = −0.757; *p* = 0.449), income (Z = −0.006; *p* = 0.995), self-assessed health status (Z = −0.325; *p* = 0.745) and self-assessed health self-care (Z = 1.236; *p* = 0.216).

### 3.3. Micronutrient Intake Adequacy

Micronutrient intake is shown by dietary groups (white), with the groups further subdivided into DS users (dark grey) and nonusers (light grey). For clearer representation, data was divided in multiple tables: vitamins A and D (Table 2), vitamins E, K and C (Table 3), thiamin, riboflavin and niacin (Table 4), pantothenic acid, pyridoxine and biotin (Table 5), folate, Vitamin B12 and potassium (Table 6), calcium, phosphorus and iron (Table 7), magnesium, iodine and zinc (Table 8), selenium, copper and manganese (Table 9) and chromium, molybdenum, sodium and chloride (Table 10). The intake with food alone is presented for DS nonusers, while for the DS users, the intake with food alone (middle grey) and intake with food plus DS (dark grey) is shown (Table 2, Table 3, Table 4, Table 5, Table 6, Table 7, Table 8, Table 9 and Table 10). Vitamin D is shown in two parts (Table 2); the first part shows all participants and the second just the participants who were measured in wintertime. We analyzed wintertime participants separately because Slovenia is on a latitude where sun exposure from October to March is not sufficient for endogenous synthesis of vitamin D, due to the angle of the sun, and therefore vitamin D supplementation is recommended.

Between-group differences in micronutrient intake with food alone of all participants from a dietary group (DS nonusers and DS users) were analyzed and the prevalence of intake adequacy of each micronutrient is shown (Table 2, Table 3, Table 4, Table 5, Table 6, Table 7, Table 8, Table 9 and Table 10). Participants from all dietary groups had a low prevalence of adequate micronutrient intake with food alone for vitamin D, iodine, potassium, molybdenum, pantothenic acid, and calcium. Many participants did not reach the recommended intake for numerous micronutrients, despite the adequate mean intake of the group. For example, mean vitamin A intake in the vegan group was 1.2 mg and in the LCHF group it was 1.0 mg, but only 53% of vegans and 54% of the LCHF group met the vitamin A RV, which is 1.0 mg [24]. Furthermore, mean pantothenic acid intake in the LCHF group was 7 mg, but only half of the group met the RV of 6 mg [24]. Vegans had the highest mean intake of potassium, with 4100 mg (the RV in Slovenia is 4000 mg [24]), but only 38% met the RV.

Differences in the prevalence of DS users between dietary groups were analyzed (Table 2, Table 3, Table 4, Table 5, Table 6, Table 7, Table 8, Table 9 and Table 10). The statistical difference between DS users and nonusers was observed in the vegetarian group for calcium intake, where DS users had higher calcium intake with food alone than nonusers (Z = −2.310; *p* = 0.021). Despite no significant difference in mean intake, a higher prevalence of adequate intake with food alone was seen in vegetarian DS users for vitamin E (Z = −2.203; *p* = 0.028). Other differences between DS users and nonusers in intakes with food alone were not observed.

**Table 2:** Vitamins A and D intake and prevalence (%) of participants with adequate intake with food alone and with inclusion of dietary supplements.

Variable (Unit)			All (M ± SD)	Omnivorous (M ± SD)	Vegan (M ± SD)	Vegetarian (M ± SD)	LCHF (M ± SD)	RV ^1^
Vitamin A (mg)	All *	Food sources	0.9 ± 0.8	0.8 ± 0.4 ^c^	1.2 ± 1.4 ^d^	0.8 ± 0.4 ^f^	1.0 ± 0.4	1.0
		Adequate intake (%)	41.5%	29.7%	53.1%	35.1%	54.2%	
	Nonusers	Food sources	0.9 ± 0.8	0.8 ± 0.4	1.2 ± 1.5	0.7 ± 0.3	1.1 ± 0.5	
		Adequate intake (%)	41.2%	27.8%	55.2%	30.3%	68.8%	
	DS users ^+^		12.3% (N = 16)	2.7% (N = 1) ^c^	9.4% (N = 3) ^e^	10.8% (N = 4) ^f^	33.3% (N = 8)	
		Food sources	0.9 ± 0.5	1.3	0.7 ± 0.3	1.3 ± 0.8	0.8 ± 0.1	
		Adequate intake (%)	43.8%	100%	33.3%	75%	25%	
		Food sources + DS	1.6 ± 0.5	1.7	1.4 ± 0.6	1.7 ± 0.8	1.6 ± 0.4	
		Adequate intake (%)	87.5%	100%	66.7%	75%	100%	
Vitamin D (µg)	All *	Food sources	4 ± 4	5 ± 4 ^a,b,c^	2 ± 2 ^d,e^	3 ± 3 ^f^	7 ± 4	20
		Adequate intake (%)	0%	0%	0%	0%	0%	
	Nonusers	Food sources	4 ± 4	5 ± 4	2 ± 2	3 ± 3	8 ± 4	
		Adequate intake (%)	0%	0%	0%	0%	0%	
	DS users		33.8% (N = 44)	18.9% (N = 7)	43.8% (N = 14)	35.1% (N = 13)	41.7% (N = 10)	
		Food sources	3 ± 4	6 ± 5	1 ± 1	3 ± 3	5 ± 2	
		Adequate intake (%)	0%	0%	0%	0%	0%	
		Food sources + DS	41 ± 41	39 ± 24	38 ± 22	58 ± 67	22 ± 15	
		Adequate intake (%)	68.2%	71.4%	85.7%	69.2%	40.0%	
Vitamin D (µg)—winter	All *	Food sources	3 ± 3	4 ± 3 ^a^	1 ± 2 ^e^	3 ± 4 ^f^	5 ± 2	20
	N = 51	Adequate intake (%)	0%	0%	0%	0%	0%	
	Nonusers		56.9% (N = 29)	88.9% (N = 16)	36.4% (N = 4)	47.1% (N = 8)	20.0% (N = 1)	
		Food sources	3 ± 3	3 ± 2	2 ± 2	4 ± 4	7	
		Adequate intake (%)	0%	0%	0%	0%	0%	
	DS users ^+^		43.1% (N = 22)	11.1% (N = 2) ^a,b,c^	63.6% (N = 7)	52.9% (N = 9)	80.0% (N = 4)	
		Food sources	3 ± 4	8 ± 6	1 ± 1	3 ± 3	5 ± 2	
		Adequate intake (%)	0%	0%	0%	0%	0%	
		Food sources + DS	46 ± 53	16 ± 6	37 ± 13	69 ± 78	26 ± 10	
		Adequate intake (%)	77.3%	50.0%	100%	66.7%	75.0%	

LCHF—low-carbohydrate high-fat; DS—dietary supplement; RV—Reference Values for Slovene population. * *p* < 0.05, Kruskal-Wallis H test of micronutrient intake with food alone for all participants between dietary groups; ^+^ *p* < 0.05, Kruskal-Wallis H test of DS use prevalence between dietary groups; ^a–f^—*p* < 0.05, Mann-Whitney test between: ^a^—omnivorous and vegan group; ^b^—omnivorous and vegetarian group; ^c^—omnivorous and LCHF group; ^d^—vegan and vegetarian group; ^e^—vegan and LCHF group; ^f^—vegetarian and LCHF group. ^1^—[24].

**Table 3:** Vitamins E, K and C intake and prevalence (%) of participants with adequate intake with food alone and with inclusion of dietary supplements.

Variable (Unit)			All (M ± SD)	Omnivorous (M ± SD)	Vegan (M ± SD)	Vegetarian (M ± SD)	LCHF (M ± SD)	RV ^1^
Vitamin E (mg)	All *	Food sources	13 ± 7	12 ± 7 ^c^	13 ± 5	13 ± 8	16 ± 7	13–15
		Adequate intake (%)	45.4%	37.8%	50.0%	35.1%	66.7%	
	Nonusers	Food sources	13 ± 7	12 ± 8	13 ± 5	13 ± 8	17 ± 8	
		Adequate intake (%)	41.0%	37.1%	50.0%	26.7%	66.7%	
	DS users ^+^		19.2% (N = 25)	5.4% (N = 2) ^c^	12.5% (N = 4) ^e^	18.9% (N = 7) ^f^	50.0% (N = 12)	
		Food sources	14 ± 5	12 ± 2	13 ± 5	15 ± 4	14 ± 6	
		Adequate intake (%)	64.0%	50.0%	50.0%	71.4%	66.7%	
		Food sources + DS	37 ± 54	27 ± 17	90 ± 140	24 ± 7	28 ± 11	
		Adequate intake (%)	96.0%	100%	100%	100%	91.7%	
Vitamin K (µg)	All *	Food sources	200 ± 170	200 ± 160	280 ± 240 ^d,e^	160 ± 80	160 ± 150	70–80
		Adequate intake (%)	85.4%	81.1%	93.8%	94.6%	66.7%	
	Nonusers	Food sources	190 ± 170	180 ± 130	290 ± 260	150 ± 80	160 ±160	
		Adequate intake (%)	85.2%	80.6%	96.3%	93.8%	65%	
	DS users		11.5% (N = 15)	2.7% (N = 1)	15.6% (N = 5)	13.5% (N = 5)	16.7% (N = 4)	
		Food sources	250 ± 180	774	250 ± 150	220 ± 80	140 ± 90	
		Adequate intake (%)	86.7%	100%	80.0%	100%	75.0%	
		Food sources + DS	280 ± 180	789	280 ± 160	280 ± 60	170 ± 100	
		Adequate intake (%)	100%	100%	100%	100%	100%	
Vitamin C (mg)	All *	Food sources	140 ± 120	100 ± 60 ^a,b^	200 ± 180 ^d,e^	150 ± 100	110 ± 70	110
		Adequate intake (%)	58.5%	40.5%	78.1%	64.9%	50.0%	
	Nonusers	Food sources	130 ± 80	90 ± 50	180 ± 90	140 ± 100	130 ± 70	
		Adequate intake (%)	57.9%	36.7%	78.3%	63.3%	58.3%	
	DS users ^+^		26.9% (N = 35)	18.9% (N = 7) ^c^	28.1% (N = 9)	18.9% (N = 7) ^f^	50.0% (N = 12)	
		Food sources	160 ± 180	150 ± 100	260 ± 320	160 ± 110	90 ± 50	
		Adequate intake (%)	60.0%	57.1%	77.8%	71.4%	41.7%	
		Food sources + DS	520 ± 860	280 ± 120	900 ± 1600	520 ± 460	370 ± 320	
		Adequate intake (%)	91.4%	100%	100%	85.7%	83.3%	

LCHF—low-carbohydrate high-fat; DS—dietary supplement; RV—Reference Values for Slovene population. * *p* < 0.05, Kruskal-Wallis H test of micronutrient intake with food alone for all participants between dietary groups; ^+^ *p* < 0.05, Kruskal-Wallis H test of DS use prevalence between dietary groups; ^a–f^—*p* < 0.05, Mann-Whitney test between: ^a^—omnivorous and vegan group; ^b^—omnivorous and vegetarian group; ^c^—omnivorous and LCHF group; ^d^—vegan and vegetarian group; ^e^—vegan and LCHF group; ^f^—vegetarian and LCHF group. ^1^—[24].

**Table 4:** Thiamin, riboflavin and niacin intake and prevalence (%) of participants with adequate intake with food alone and with inclusion of dietary supplements.

Variable (Unit)			All (M ± SD)	Omnivorous (M ± SD)	Vegan (M ± SD)	Vegetarian (M ± SD)	LCHF (M ± SD)	RV ^1^
Thiamin (mg)	All *	Food sources	1.3 ± 0.7	1.3 ± 0.5 ^c^	1.6 ± 0.9 ^d,e^	1.3 ± 0.6	1.0 ± 0.6	1.2–1.3
		Adequate intake (%)	60.8%	67.6%	71.9%	59.5%	37.5%	
	Nonusers	Food sources	1.4 ± 0.7	1.3 ± 0.4	1.7 ± 1.0	1.3 ± 0.6	1.0 ± 0.6	
		Adequate intake (%)	62.4%	68.6%	70.8%	58.6%	38.5%	
	DS users ^+^		22.3% (N = 29)	5.4% (N = 2) ^a,b,c^	25.0% (N = 8)	21.6% (N = 8) ^f^	45.8% (N = 11)	
		Food sources	1.3 ± 0.5	1.9 ± 1.4	1.4 ± 0.4	1.2 ± 0.4	1.1 ± 0.5	
		Adequate intake (%)	55.2%	50.0%	75.0%	62.5%	36.4%	
		Food sources + DS	4.7 ± 6.0	3.7 ± 0.3	3.3 ± 1.3	3.8 ± 1.9	6.5 ± 9.5	
		Adequate intake (%)	96.6%	100%	100%	100%	90.9%	
Riboflavin (mg)	All *	Food sources	1.6 ± 0.7	1.7 ± 0.6 ^a,b,c^	1.4 ± 0.6 ^e^	1.4 ± 0.7 ^f^	2.0 ± 0.7	1.3–1.4
		Adequate intake (%)	82.3%	91.9%	65.5%	75.7%	100%	
	Nonusers	Food sources	1.6 ± 0.7	1.7 ± 0.5	1.4 ± 0.7	1.4 ± 0.7	2.1 ± 0.8	
		Adequate intake (%)	80.0%	91.4%	62.5%	71.4%	100%	
	DS users ^+^		23.1% (N = 30)	5.4% (N = 2) ^a,b,c^	25.0% (N = 8)	24.3% (N = 9)	45.8% (N = 11)	
		Food sources	1.6 ± 0.6	2.3 ± 1.3	1.3 ± 0.5	1.6 ± 0.8	1.8 ± 0.4	
		Adequate intake (%)	90.0%	100%	75.0%	88.9%	100%	
		Food sources + DS	5.1 ± 6.0	4.5 ± 0.7	3.4 ± 1.5	4.5 ± 2.3	7.0 ± 9.5	
		Adequate intake (%)	100%	100%	100%	100%	100%	
Niacin (mg)	All *	Food sources	18 ± 9	24 ± 10 ^a,b,c^	15 ± 7	17 ± 7	15 ± 7	15–16
		Adequate intake (%)	70.8%	91.9%	59.4%	75.7%	45.8%	
	Nonusers	Food sources	18 ± 9	23 ± 9	15 ± 8	17 ± 8	14 ± 7	
		Adequate intake (%)	73.8%	91.4%	58.3%	80.0%	42.9%	
	DS users ^+^		20.8% (N = 27)	5.4% (N = 2) ^a,c^	25.0% (N = 8)	18.9% (N = 7)	41.7% (N = 10)	
		Food sources	17 ± 11	47 ± 11	14 ± 5	17 ± 8	15 ± 7	
		Adequate intake (%)	59.3%	100%	62.5%	57.1%	50.0%	
		Food sources + DS	35 ± 14	67 ± 4	30 ± 9	30 ± 15	37 ± 10	
		Adequate intake (%)	100%	100%	100%	100%	100%	

LCHF—low-carbohydrate high-fat; DS—dietary supplement; RV—Reference Values for Slovene population. * *p* < 0.05, Kruskal-Wallis H test of micronutrient intake with food alone for all participants between dietary groups; ^+^ *p* < 0.05, Kruskal-Wallis H test of DS use prevalence between dietary groups; ^a–f^—*p* < 0.05, Mann-Whitney test between: ^a^—omnivorous and vegan group; ^b^—omnivorous and vegetarian group; ^c^—omnivorous and LCHF group; ^d^—vegan and vegetarian group; ^e^—vegan and LCHF group; ^f^—vegetarian and LCHF group. ^1^—[24].

**Table 5:** Pantothenic acid, pyridoxine and biotin intake and prevalence (%) of participants with adequate intake with food alone and with inclusion of dietary supplements.

Variable (Unit)			All (M ± SD)	Omnivorous (M ± SD)	Vegan (M ± SD)	Vegetarian (M ± SD)	LCHF (M ± SD)	RV ^1^
Pantothenic acid (mg)	All *	Food sources	5 ± 3	5 ± 2 ^c^	6 ± 4 ^e^	5 ± 2 ^f^	7 ± 3	6
		Adequate intake (%)	30.8%	21.6%	28.1%	29.7%	50.0%	
	Nonusers	Food sources	5 ± 3	5 ± 2	5 ± 3	5 ± 3	7 ± 3	
		Adequate intake (%)	30.8%	20.0%	26.9%	34.5%	57.1%	
	DS users ^+^		20.0% (N = 26)	5.4% (N = 2) ^b,c^	18.8% (N = 6)	21.6% (N = 8)	41.7% (N = 10)	
		Food sources	6 ± 5	8 ± 4	8 ± 8	4 ± 2	6 ± 2	
		Adequate intake (%)	30.8%	50.0%	33.3%	12.5%	40.0%	
		Food sources + DS	14 ± 8	17 ± 5	13 ± 9	12 ± 8	16 ± 9	
		Adequate intake (%)	92.3%	100%	100%	75.0%	100%	
Pyridoxine (mg)	All	Food sources	2.3 ± 2.3	2.3 ± 1.7	2.4 ± 1.8	2.5 ± 3.6	1.7 ± 0.7	1.6
		Adequate intake (%)	68.5%	70.3%	81.3%	62.2%	58.3%	
	Nonusers	Food sources	2.2 ± 1.6	2.0 ± 1.0	2.7 ± 2.0	2.1 ± 2.0	2.0 ± 0.8	
		Adequate intake (%)	69.3%	68.6%	87.0%	60.0%	61.5%	
	DS users ^+^		22.3% (N = 29)	5.4 (N = 2) ^a,c^	28.1% (N = 9)	18.9% (N = 7) ^f^	45.8% (N = 11)	
		Food sources	2.6 ± 4.0	7.1 ± 4.4	1.8 ± 0.7	4.4 ± 7.4	1.3 ± 0.5	
		Adequate intake (%)	65.5%	100%	66.7%	71.4%	54.5%	
		Food sources + DS	10.0 ± 19.7	9.7 ± 6.6	15.9 ± 33.4	7.9 ± 8.9	6.5 ± 9.6	
		Adequate intake (%)	100%	100%	100%	100%	100%	
Biotin (µg)	All *	Food sources	40 ± 26	37 ± 16 ^a,c^	30 ± 25 ^e^	32 ± 17 ^f^	70 ± 29	30–60
		Adequate intake (%)	55.4%	56.8%	28.1%	48.6%	100%	
	Nonusers	Food sources	41 ± 27	37 ± 15	32 ± 28	32 ± 17	82 ± 33	
		Adequate intake (%)	54.9%	55.9%	37.5%	46.7%	100%	
	DS users ^+^		21.5% (N = 28)	8.1% (N = 3) ^c^	25.0% (N = 8)	18.9% (N = 7)	41.7% (N = 10)	
		Food sources	39 ± 19	47 ± 24	23 ± 7	33 ± 18	54 ± 13	
		Adequate intake (%)	57.1%	66.7%	0%	57.1%	100%	
		Food sources + DS	120 ± 110	320 ± 180	68 ± 34	120 ± 120	100 ± 4	
		Adequate intake (%)	92.9%	100%	75.0%	100%	100%	

LCHF—low-carbohydrate high-fat; DS—dietary supplement; RV—Reference Values for Slovene population. * *p* < 0.05, Kruskal-Wallis H test of micronutrient intake with food alone for all participants between dietary groups; ^+^ *p* < 0.05, Kruskal-Wallis H test of DS use prevalence between dietary groups; ^a–c,e,f^—*p* < 0.05, Mann-Whitney test between: ^a^—omnivorous and vegan group; ^b^—omnivorous and vegetarian group; ^c^—omnivorous and LCHF group; ^e^—vegan and LCHF group; ^f^—vegetarian and LCHF group. ^1^—[24].

**Table 6:** Folate, Vitamin B_12_ and potassium intake and prevalence (%) of participants with adequate intake with food alone and with inclusion of dietary supplements.

Variable (Unit)			All (M ± SD)	Omnivorous (M ± SD)	Vegan (M ± SD)	Vegetarian (M ± SD)	LCHF (M ± SD)	RV ^1^
Folate (µg)	All *	Food sources	380 ± 260	340 ± 130 ^a^	530 ± 430 ^d,e^	360 ± 150	290 ± 120	300
		Adequate intake (%)	58.5%	51.4%	81.3%	56.8%	41.7%	
	Nonusers	Food sources	400 ± 280	340 ± 120	580 ± 490	370 ± 160	330 ± 130	
		Adequate intake (%)	62.4%	52.9%	87.0%	55.2%	60.0%	
	DS users ^+^		22.3% (N = 29)	8.1% (N = 3) ^a,c^	28.1% (N = 9)	21.6% (N = 8)	37.5% (N = 9)	
		Food sources	330 ± 160	330 ± 260	430 ± 180	330 ± 90	240 ± 100	
		Adequate intake (%)	44.8%	33.3%	66.7%	62.5%	11.1%	
		Food sources + DS	610 ± 260	540 ± 200	730 ± 300	610 ± 330	510 ± 160	
		Adequate intake (%)	100%	100%	100%	100%	100%	
Vitamin B_12_ (µg)	All *	Food sources	10 ± 73	4.3 ± 2.9 ^a,b,c^	27 ± 147 ^d,e^	3.2 ± 4.2 ^f^	7.0 ± 6.3	4.0
		Adequate intake (%)	33.8%	43.2%	6.3%	16.2%	83.3%	
	Nonusers	Food sources	4.1 ± 4.3	4.0 ± 2.6	0.8 ± 0.9	2.8 ± 2.9	8.1 ± 7.4	
		Adequate intake (%)	37.8%	40.0%	0%	15.4%	92.9%	
	DS users ^+^		36.9% (N = 48)	5.4% (N = 2) ^a,b,c^	78.1% (N = 25) ^d,e^	29.7% (N = 11)	41.7% (N = 10)	
		Food sources	20 ± 120	9.5 ± 3.2	35 ± 170	4.3 ± 6.3	5.6 ± 4.0	
		Adequate intake (%)	27.1%	100%	8.0%	18.2%	70.0%	
		Food sources + DS	420 ± 830	14.3 ± 9.2	670 ± 1050	250 ± 540	90 ± 160	
		Adequate intake (%)	89.6%	100%	84.0%	90.9%	100%	
Potassium (mg)	All *	Food sources	3200 ± 1800	3200 ± 1100 ^c^	4100 ± 2900 ^e^	3100 ± 1100 ^f^	2500 ± 700	4000
		Adequate intake (%)	22.3%	18.9%	37.5%	21.6%	8.3%	
	Nonusers	Food sources	3200 ± 1800	3100 ± 1000	4100 ± 2900	3100 ± 1100	2400 ± 700	
		Adequate intake (%)	22.0%	16.7%	37.5%	21.6%	9.1%	
	DS users		2.3% (N = 3)	2.7% (N = 1)	0%	0%	8.3% (N = 2)	
		Food sources	4300 ± 2100	6600	/	/	3100 ± 700	
		Adequate intake (%)	33.3%	100%	/	/	0%	
		Food sources + DS	4300 ± 2100	6700	/	/	3100 ± 700	
		Adequate intake (%)	33.3%	100%	/	/	0%	

LCHF—low-carbohydrate high-fat; DS—dietary supplement; RV—Reference Values for Slovene population. * *p* < 0.05, Kruskal-Wallis H test of micronutrient intake with food alone for all participants between dietary groups; ^+^ *p* < 0.05, Kruskal-Wallis H test of DS use prevalence between dietary groups; ^a–f^—p < 0.05, Mann-Whitney test between: ^a^—omnivorous and vegan group; ^b^—omnivorous and vegetarian group; ^c^—omnivorous and LCHF group; ^d^—vegan and vegetarian group; ^e^—vegan and LCHF group; ^f^—vegetarian and LCHF group. ^1^—[24].

**Table 7:** Calcium, phosphorus and iron intake and prevalence (%) of participants with adequate intake with food alone and with inclusion of dietary supplements.

Variable (Unit)			All (M ± SD)	Omnivorous (M ± SD)	Vegan (M ± SD)	Vegetarian (M ± SD)	LCHF (M ± SD)	RV ^1^
Calcium (mg)	All	Food sources	840 ± 380	960 ± 370	730 ± 310	810 ± 350	870 ± 480	1000
		Adequate intake (%)	26.9%	43.2%	12.5%	24.3%	25.0%	
	Nonusers	Food sources	820 ± 380	940 ± 360	740 ± 310	750 ± 300	870 ± 550	
		Adequate intake (%)	23.9%	41.7%	12.9%	15.9%	22.1%	
	DS users ^+^		10.0% (N = 13)	2.7% (N = 1) ^c^	3.1% (N = 1) ^e^	13.5% (N = 5)	25.0% (N = 6)	
		Food sources	1020 ± 380	1570	590	1200 ± 420	850 ± 210	
		Adequate intake (%)	53.8%	100%	0%	80.0%	33.2%	
		Food sources + DS	1200 ± 410	1630	640	1390 ± 380	1070 ± 350	
		Adequate intake (%)	61.5%	100%	0%	80.0%	50.0%	
Phosphorus (mg)	All *	Food sources	1230 ± 470	1350 ± 440 ^a,b^	1120 ± 560 ^e^	1100 ± 370 ^f^	1430 ± 450	700
		Adequate intake (%)	90.0%	94.6%	78.1%	89.2%	100%	
	Nonusers	Food sources	1230 ± 460	1310 ± 390	1130 ± 560	1110 ± 370	1440 ± 480	
		Adequate intake (%)	89.3%	94.4%	77.4%	88.6%	100%	
	DS users		6.2% (N = 8)	2.7% (N = 1)	3.1% (N = 1)	5.4% (N = 2)	16.7% (N = 4)	
		Food sources	1320 ± 600	2580	750	900 ± 240	1360 ± 270	
		Adequate intake (%)	100%	100%	100%	100%	100%	
		Food sources + DS	1450 ± 620	2640	780	1020 ± 220	1540 ± 370	
		Adequate intake (%)	100%	100%	100%	100%	100%	
Iron (mg) ^2^	All *	Food sources	17 ± 10	16 ± 6 ^a^	22 ± 15 ^d,e^	16 ± 7	14 ± 5	10
		Adequate intake (%)	90.0%	91.9%	96.9%	83.8%	87.5%	
	Nonusers	Food sources	17 ± 10	16 ± 6	23 ± 16	16 ± 7	14 ± 6	
		Adequate intake (%)	89.3%	91.2%	96.6%	83.9%	83.3%	
	DS users		13.8% (N = 18)	8.1% (N = 3)	9.4% (N = 3)	16.2% (N = 6)	25.0% (N = 6)	
		Food sources	17 ± 6	17 ± 11	20 ± 6	18 ± 5	15 ± 3	
		Adequate intake (%)	94.4%	100%	100%	83.3%	100%	
		Food sources + DS	29 ± 12	23 ± 9	34 ± 22	28 ± 8	31 ± 13	
		Adequate intake (%)	100%	100%	100%	100%	100%	

LCHF—low-carbohydrate high-fat; DS—dietary supplement; RV—Reference Values for Slovene population. * *p* < 0.05, Kruskal-Wallis H test of micronutrient intake with food alone for all participants between dietary groups; ^+^ *p* < 0.05, Kruskal-Wallis H test of DS use prevalence between dietary groups; ^a–f^—*p* < 0.05, Mann-Whitney test between: ^a^—omnivorous and vegan group; ^b^—omnivorous and vegetarian group; ^c^—omnivorous and LCHF group; ^d^—vegan and vegetarian group; ^e^—vegan and LCHF group; ^f^—vegetarian and LCHF group. ^1^—[24]. ^2^ The amount of iron intake was not corrected for lower bioavailability for those on a plant-based diet.

**Table 8:** Magnesium, iodine and zinc intake and prevalence (%) of participants with adequate intake with food alone and with inclusion of dietary supplements.

Variable (Unit)			All (M ± SD)	Omnivorous (M ± SD)	Vegan (M ± SD)	Vegetarian (M ± SD)	LCHF (M ± SD)	RV ^1^
Magnesium (mg)	All *	Food sources	420 ± 270	390 ± 180 ^a^	560 ± 390 ^d,e^	410 ± 230	290 ± 130	350–400
		Adequate intake (%)	61.5%	54.1%	81.3%	64.9%	41.7%	
	Nonusers	Food sources	440 ± 290	370 ± 160	590 ± 420	440 ± 250	280 ± 140	
		Adequate intake (%)	62.6%	50.0%	85.7%	66.7%	33.3%	
	DS users ^+^		23.8% (N = 31)	13.5% (N = 5) ^c^	12.5% (N = 4) ^e^	27.0% (N = 10)	50.0% (N = 12)	
		Food sources	360 ± 170	520 ± 240	400 ± 140	340 ± 140	290 ± 120	
		Adequate intake (%)	58.1%	80.0%	50.0%	60.0%	50.0%	
		Food sources + DS	710 ± 540	1300 ± 1200	670 ± 260	570 ± 160	590 ± 250	
		Adequate intake (%)	93.5%	100%	100%	90.0%	91.7%	
Iodine (µg)	All *	Food sources	80 ± 50	90 ± 40	70 ± 50 ^e^	80 ± 40	100 ± 60	180–200
		Adequate intake (%)	3.1%	2.7%	3.1%	0%	8.3%	
	Nonusers	Food sources	80 ± 50	90 ± 40	70 ± 50	70 ± 40	100 ± 60	
		Adequate intake (%)	3.4%	2.8%	3.1%	0%	11.1%	
	DS users ^+^		8.5 % (N = 11)	2.7 % (N = 1) ^c^	0% (N = 0) ^e^	10.8% (N = 4)	25.0% (N = 6)	
		Food sources	100 ± 40	160	/	110 ± 40	90 ± 40	
		Adequate intake (%)	0%	0%	/	0%	0%	
		Food sources + DS	190 ± 50	190	/	180 ± 60	190 ± 60	
		Adequate intake (%)	63.6%	100%	/	50.0%	66.7%	
Zinc (mg)	All *	Food sources	10 ± 5	11 ± 5 ^b^	10 ± 6 ^e^	9 ± 5 ^f^	11 ± 4	11–16
		Adequate intake (%)	62.3%	70.3%	59.4%	45.9%	79.2%	
	Nonusers	Food sources	10 ± 5	11 ± 4	9 ± 5	9 ± 4	11 ± 4	
		Adequate intake (%)	62.2%	71.4%	60.7%	48.5%	73.3%	
	DS users ^+^		14.6% (N = 19)	5.4% (N = 2) ^c^	12.5% (N = 4) ^e^	10.8% (N = 4) ^f^	37.5% (N = 9)	
		Food sources	12 ± 7	12 ± 9	14 ± 10	13 ± 12	11 ± 3	
		Adequate intake (%)	63.2%	50.0%	50.0%	25.0%	88.9%	
		Food sources + DS	24 ± 15	14 ± 9	33 ± 27	19 ± 11	25 ± 11	
		Adequate intake (%)	94.7%	100%	75.0%	100%	100%	

LCHF—low-carbohydrate high-fat; DS—dietary supplement; RV—Reference Values for Slovene population. * *p* < 0.05, Kruskal-Wallis H test of micronutrient intake with food alone for all participants between dietary groups; ^+^ *p* < 0.05, Kruskal-Wallis H test of DS use prevalence between dietary groups; ^a–f^—*p* < 0.05, Mann-Whitney test between: ^a^—omnivorous and vegan group; ^b^—omnivorous and vegetarian group; ^c^—omnivorous and LCHF group; ^d^—vegan and vegetarian group; ^e^—vegan and LCHF group; ^f^—vegetarian and LCHF group. ^1^—[24].

**Table 9:** Selenium, copper and manganese intake and prevalence (%) of participants with adequate intake with food alone and with inclusion of dietary supplements.

Variable (Unit)			All (M ± SD)	Omnivorous (M ± SD)	Vegan (M ± SD)	Vegetarian (M ± SD)	LCHF (M ± SD)	RV ^1^
Selenium (µg)	All *	Food sources	90 ± 80	110 ± 120 ^a,b^	60 ± 30 ^e^	75 ± 60 ^f^	120 ± 60	M: 70
		Adequate intake (%)	61.5%	75.7%	37.5%	48.6%	91.7%	F: 60
	Nonusers	Food sources	90 ± 80	110 ± 110	60 ± 40	70 ± 60	120 ± 60	
		Adequate intake (%)	59.0%	75.0%	35.5%	47.1%	93.8%	
	DS users ^+^		10.0% (N = 13)	2.7% (N = 1) ^c^	3.1% (N = 1) ^e^	8.1% (N = 3) ^f^	33.3% (N = 8)	
		Food sources	120 ± 80	310	70	90 ± 40	110 ± 60	
		Adequate intake (%)	84.6%	100%	100%	66.7%	87.5%	
		Food sources + DS	140 ± 70	330	80	110 ± 50	135 ± 50	
		Adequate intake (%)	100%	100%	100%	100%	100%	
Copper (mg)	All *	Food sources	2.0 ± 1.1	1.9 ± 0.7 ^a,c^	2.7 ± 1.7 ^d,e^	1.9 ± 0.8 ^f^	1.4 ± 0.7	1.0–1.5
		Adequate intake (%)	90.0%	91.9%	100%	94.6%	66.7%	
	Nonusers	Food sources	2.0 ± 1.1	1.8 ± 0.7	2.7 ± 1.7	1.9 ± 0.8	1.3 ± 0.6	
		Adequate intake (%)	90.1%	91.7%	100%	94.1%	65.0%	
	DS users		6.9% (N = 9)	2.7% (N = 1)	3.1% (N = 1)	8.1% (N = 3)	16.7% (N = 4)	
		Food sources	2.1 ± 0.9	3.5	2.0	2.3 ± 0.6	1.7 ± 1.0	
		Adequate intake (%)	88.9%	100%	100%	100%	75.0%	
		Food sources + DS	2.7 ± 0.9	4.0	2.2	2.8 ± 0.5	2.5 ± 1.2	
		Adequate intake (%)	100%	100%	100%	100%	100%	
Manganese (mg)	All *	Food sources	5.4 ± 3.8	5.0 ± 3.1 ^a,c^	7.7 ± 4.5 ^d,e^	5.6 ± 3.4 ^f^	2.4 ± 1.4	2.0–5.0
		Adequate intake (%)	89.2%	94.6%	100%	97.3%	54.2%	
	Nonusers	Food sources	5.5 ± 3.8	4.9 ± 3.1	7.8 ± 4.6	5.7 ± 3.4	2.3 ± 1.1	
		Adequate intake (%)	90.8%	94.4%	100%	97.1%	55.6%	
	DS users ^+^		7.7% (N = 10)	2.7% (N = 1) ^c^	3.1% (N = 1) ^e^	5.4% (N = 2) ^f^	25.0% (N = 6)	
		Food sources	4.0 ± 2.6	8.3	6.3	4.3 ± 2.6	2.8 ± 2.1	
		Adequate intake (%)	70.0%	100%	100%	100%	50.0%	
		Food sources + DS	6.1 ± 2.2	8.8	6.3	5.1 ± 2.4	5.9 ± 2.4	
		Adequate intake (%)	90.0%	100%	100%	100%	83.3%	

LCHF—low-carbohydrate high-fat; DS—dietary supplement; RV—Reference Values for Slovene population. * *p* < 0.05, Kruskal-Wallis H test of micronutrient intake with food alone for all participants between dietary groups; ^+^ *p* < 0.05, Kruskal-Wallis H test of DS use prevalence between dietary groups; ^a–f^—*p* < 0.05, Mann-Whitney test between: ^a^—omnivorous and vegan group; ^b^—omnivorous and vegetarian group; ^c^—omnivorous and LCHF group; ^d^—vegan and vegetarian group; ^e^—vegan and LCHF group; ^f^—vegetarian and LCHF group. ^1^—[24].

**Table 10:** Chromium, molybdenum, sodium and chloride intake and prevalence (%) of participants with adequate intake with food alone and with inclusion of dietary supplements.

Variable (Unit)			All (M ± SD)	Omnivorous (M ± SD)	Vegan (M ± SD)	Vegetarian (M ± SD)	LCHF (M ± SD)	RV ^1^
Chromium (µg)	All	Food sources	40 ± 50	50 ± 70	40 ± 50	40 ± 40	30 ± 30	30–100
		Adequate intake (%)	50.0%	56.8%	37.5%	54.1%	50.0%	
	Nonusers	Food sources	40 ± 50	50 ± 70	40 ± 50	40 ± 40	30 ± 20	
		Adequate intake (%)	49.1%	55.6%	41.4%	52.9%	58.8%	
	DS users ^+^		10.8% (N = 14)	2.7% (N = 1) ^c^	9.4% (N = 3)	8.1% (N = 3) ^f^	29.2% (N = 7)	
		Food sources	60 ± 60	240	20 ± 10	50 ± 50	40 ± 30	
		Adequate intake (%)	57.1%	100%	0%	66.7%	71.4%	
		Food sources + DS	80 ± 60	250	50 ± 20	70 ± 50	70 ± 30	
		Adequate intake (%)	85.7%	100%	66.7%	100%	85.7%	
Molybdenum (µg)	All	Food sources	50 ± 50	40 ± 40	55 ± 50	60 ± 60	50 ± 40	50–100
		Adequate intake (%)	30.0%	16.2%	37.5%	35.1%	33.3%	
	Nonusers	Food sources	50 ± 50	40 ± 40	55 ± 50	60 ± 60	60 ± 50	
		Adequate intake (%)	30.0%	13.9%	38.7%	34.3%	38.9%	
	DS users ^+^		7.7% (N = 10)	2.7% (N = 1) ^c^	3.1% (N = 1) ^e^	5.4% (N = 2) ^f^	25.0% (N = 6)	
		Food sources	40 ± 20	50	30	40 ± 40	30 ± 20	
		Adequate intake (%)	30.0%	100%	0%	50.0%	16.7%	
		Food sources + DS	60 ± 30	70	40	80 ± 70	60 ± 30	
		Adequate intake (%)	50.0%	100%	0%	50.0%	50.0%	
^2^ Sodium (mg)		Food sources	2400 ± 1100	2650 ± 1000	2400 ± 1200	2200 ± 1100	2300 ± 1000	1500
^2^ Chloride (mg)		Food sources	3800 ± 1600	4200 ± 1600	3900 ± 1700	3400 ± 1600	3400 ± 1500	2300

LCHF—low-carbohydrate high-fat; DS—dietary supplement; RV—Reference Values for Slovene population. ^+^ *p* < 0.05, Kruskal-Wallis H test of DS use prevalence between dietary groups; ^c,e,f^—*p* < 0.05, Mann-Whitney test between: ^c^—omnivorous and LCHF group; ^e^—vegan and LCHF group; ^f^—vegetarian and LCHF group. ^1^—[24]. ^2^—Micronutrients used for improving taste in form of salt.

### 3.4. Micronutrient Content in Multimicronutrient Dietary Supplements (MMN DS)

**Figure 2:**
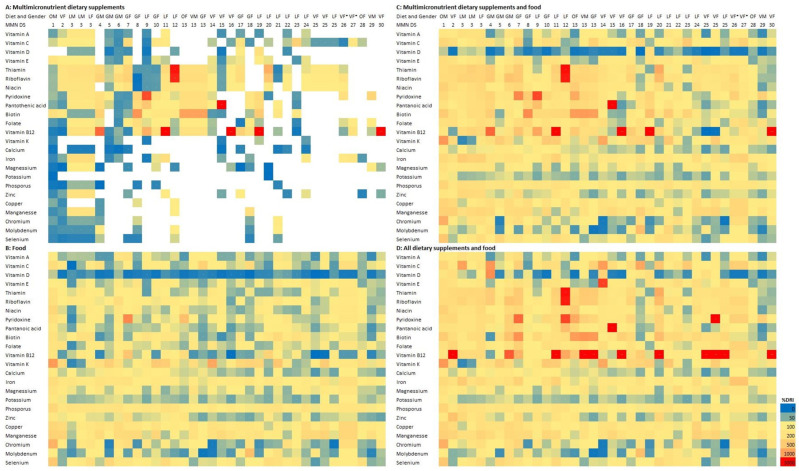
(**A**) Micronutrient content of multimicronutrient dietary supplements taken by our participants. (**B**) Micronutrient intake adequacy with food alone. (**C**) Micronutrient intake adequacy with food and multimicronutrient dietary supplements intake. (**D**) Micronutrient intake adequacy with food, multimicronutrient, and other dietary supplements intake. Diet and gender: O—omnivore; V—vegan, G—vegetarian; L—low-carbohydrate high-fat diet; M—male; F—female; MMN DS—number of multimicronutrient dietary supplements; %DRI—intake in % of daily recommended intake. * Multimicronutrient dietary supplement used by the same participant.

MMN DSs were the fourth most popular DS in our sample, as 35 (26.9%) participants took them. A total of 30 different MMN DSs were used. The content of MMN DSs was analyzed. Three MMN DSs were taken by more than one participant (Figure 2A, numbers 3, 13 and 25). One participant concomitantly took two different MMN DSs.

The most abundant components of MMN DSs were B group vitamins. MMN DSs differed in composition in terms of the vitamins and minerals they contained and in their amounts. For example, the content of riboflavin in different MMN DSs differed from 4% to 2564% of the daily RV (Figure 2A). Many participants who took MMN DSs (26; 74.3% of MMN DS users), also took other DSs at the same time.

Intake adequacy of some micronutrients with food alone was low, such as vitamin D, vitamin B_12_, calcium, and molybdenum (colored in blue, Figure 2B). Intake adequacy was increased with MMN DSs (Figure 2C), seen as a switch from blue to yellow (100% RV) mostly for B group vitamins. Intake adequacy was further improved with all added DSs (Figure 2D), seen especially in vitamin D supplementation. On the other hand, high-dose supplementation of some micronutrients was also present (red color), vitamin B_12_ was the most commonly supplemented micronutrient in high doses, even to above 3000% of the RV.

### 3.5. Tolerable Upper Intake Level

Exceeding the tolerable upper intake level (UL) was rare in our participants; 6.9% of participants exceeded the UL for niacin intake with food alone and 15.4% with included DSs. There were also individual examples of exceeding the UL with some other micronutrients, namely selenium, zinc, folate, iron, copper, pyridoxine, vitamin A, calcium, vitamin D, and vitamin C, as shown in Table 11. Vitamin E has a UL of 300 mg and molybdenum has a UL of 600 µg [25], but no participant had exceeded these levels.

**Table 11:** Prevalence of participants exceeding the tolerable upper intake level (UL) with food alone and with included dietary supplement intake.

Variable	Food Alone (%)	Food + DS (%)	UL
Niacin	6.9	15.4	35 mg ^1^
Selenium	3.1	3.1	255 µg ^2^
Zinc	1.5	6.2	25 mg ^1^
Folate	1.5	3.8	1000 µg ^1^
Iron	1.5	3.1	45 mg ^1^
Copper	1.5	1.5	5 mg ^1^
Pyridoxine	0.8	3.1	12 mg ^3^
Vitamin A	0.8	0.8	3000 µg ^1^
Calcium	0.8	0.8	2500 mg ^4^
Vitamin D	0	1.5	100 µg ^5^
Vitamin C	0	0.8	2000 mg ^1^

DS—dietary supplement; ^1^ [25], ^2^ [26], ^3^ [27], ^4^ [28], ^5^ [29].

## 4. Discussion

Micronutrient intake adequacy was analyzed in healthy adult participants with habitual omnivorous, vegan, vegetarian, and LCHF dietary patterns with normal BMI and high interest in healthy nutrition. We observed the lowest prevalence of adequate micronutrient intake with food alone for vitamin D, iodine, potassium, molybdenum, pantothenic acid, and calcium, regardless of the nutritional pattern. A low prevalence of adequate vitamin B_12_ intake was present in vegans and vegetarians and it was also relatively low in omnivores. Many participants did not reach the recommended intake for numerous micronutrients, despite adequate mean intake in the group. We observed the highest prevalence of DS use among vegans followed by those practicing LCHF, which indicates awareness of possible diet shortages, although not all insufficiencies were tackled specifically. Participants in the LCHF group took the highest number of DSs at the same time, 21% of the LCHF group took three DSs and 12% of the group took four or more DSs.

There were no significant differences in micronutrient intake with food alone between DS users and nonusers, except for calcium in the vegetarian group, which is in line with some previously reported data [19], despite some reports of higher nutrient adequacy with food alone in DS users [30]. Despite no significant difference in mean intake, a higher prevalence of adequate intake with food alone was seen in vegetarian DS users for vitamin E. We did not find any differences between DS users and nonusers in age, education, physical activity, smoking, or self-reported health, which is contrary to other studies [18,19,31], but this is probably due to our sample, which included healthy participants with normal BMI and an interest in nutrition.

### 4.1. Frequently Used DS

Vitamin B_12_ was the most popular DS, especially in the vegan group, where 78% of participants took it, which is comparable to a study from Germany [32]. Dietary intake of vitamin B_12_ was low in our vegan group and therefore supplementation was necessary. The vegetarian group had a lower prevalence of vitamin B_12_ supplementation (30%), despite the low prevalence of adequate intake of vitamin B_12_ with food alone in their group (16%), which is in line with previous findings that vegetarians are less likely to take DSs with vitamin B_12_ than vegans [7]. The highest proportion of participants with adequate vitamin B_12_ intake with food alone was in the LCHF group, followed by the omnivorous group (83% and 43%, respectively). A recent study on a Slovenian representative sample showed that 32% of the adult population did not meet the recommended vitamin B_12_ intake, but the prevalence of serum vitamin B_12_ deficiency was present was only 1.2% in the adult population [33]. Despite a high prevalence of adequate vitamin B_12_ intake with diet alone in the LCHF group, more than 40% of the participants from the group took DSs containing vitamin B_12_, the majority as part of MMN DS. We observed that many participants who a took vitamin B_12_-only supplement, with the highest proportion in the vegan group, took high doses, such as 1200 μg, 1000 μg, 500 μg, and 400 μg daily, which were doses recommended by the DS manufacturers. Such recommendations by the manufacturers exceed the maximum level for the addition of vitamin B_12_ to foods including food supplements published by official institutions; the German Federal Institute for Risk Assessment recommends the maximum level of daily recommended dose of vitamin B_12_ per DS to be 25 μg [34,35]. Some participants combined a vitamin B_12_ DS with MMN DSs that also contained vitamin B_12_ and reached daily doses as high as 5000 μg daily. The tolerable upper intake level (UL) for vitamin B_12_ is not set [36], but nevertheless there is some evidence of adverse side effects of chronic high-dose vitamin B_12_ supplementation, especially with combined high pyridoxine intake [37]. High vitamin B_12_ intake in combination with high pyridoxine intake was observed in four participants, of which one had an intake of both micronutrients above 3000% of the RV. It is known that vitamin B_12_ is better absorbed when it is regularly included in food than from a one-time high dose [38], but this does not justify the high daily dosage observed. The efficiency of absorption of vitamin B_12_ decreases with supplement dose, from 56% of a 1 μg dose to 1.3% of a 1000 μg dose [38]. Additionally, a 350 μg dosage per week distributed among 50 μg doses per day was comparable in terms of effectiveness to a single 2000 μg dose per week in restoring normal cobalamine plasma levels in vegetarians and vegans with mild deficiency [39]. This points towards such high doses of vitamin B_12_ in healthy vegetarians and vegans being needless. Moreover, a study of a Canadian representative sample showed that doses up to 10–25 μg for adults efficiently decrease the prevalence of vitamin B_12_ deficiency, whereas higher doses did not contribute further [40].

The second most popular DS was vitamin D. A total of 38% of participants took it, which is similar to a study from Germany [32], but a lot less compared to a Finnish report on vegan and omnivorous groups [41]. The highest prevalence of use of this DS was found in the vegan and LCHF groups (44% and 42%, respectively), followed by the vegetarian group and the omnivorous group (35% and 19%, respectively). The vegan group had the lowest and the LCHF group had the highest vitamin D intake with food alone, which is in line with previous reports [7,8,11], but regardless of these differences, the intakes were too low in all groups. Vitamin D can be biosynthesized internally in skin with sun exposure, but at our latitudes (above 35° north) the incident angle of the sun is too small in wintertime for the biosynthesis of vitamin D in the skin to occur [11]. In a Slovenian representative sample, a high prevalence of vitamin D deficiency was shown in the winter months and this was lower but still high in the summer months, which points to the need of vitamin D supplementation, at least in the winter months [11]. It is important to note that we do not have data regarding sun exposure of the participants that participated in the study during summer, and thus we cannot know if suitable levels were reached through photoreaction in skin. None of our participants reached adequate vitamin D intake with food alone. Only 43% of participants who participated to the study in the winter months took a DS with vitamin D and nearly one-quarter of them did not reach the recommended intake of 20 μg per day [24], despite the DS use. Only two omnivorous participants (11%) took a DS with vitamin D in winter and only one of them reached the RV. Our results point to an insufficient awareness in the population of the need of vitamin D supplementation in the winter months and a lack of knowledge regarding the suitable dose. The majority of MMN DSs reported by our participants had low doses of vitamin D (Figure 2A). In European regulation for food labeling, the RV for vitamin D is still set at 5 μg per day [42], so the relatively low doses of vitamin D in DSs appear high when expressed in %RV. Furthermore, the dose of vitamin D in DSs is often reported in international units while in the reference values for the Slovene population, the dose is expressed in μg [24]. Although the RV contains a conversion factor in the footnote, the information might not be easy to obtain and/or understand by the public. Despite relatively low vitamin D DS use in comparison to recommended supplementation DS use increased the prevalence of adequate vitamin D intake from 0 to 68% in DS users.

DSs with vitamin C were also commonly used among our participants, more than a fourth of participants took them. Not all the participants that used vitamin C DSs would need supplementation (60% of users had adequate vitamin C intake), but vitamin C supplementation increased vitamin C intake adequacy in vitamin DS users to 91%. The highest prevalence of vitamin C DS use was in the LCHF group, followed by the vegan group. The vegetarian and omnivorous groups had the lowest prevalence of vitamin C DS use, despite the fact that the omnivorous group also had the lowest prevalence of adequate vitamin C intake with food alone, which amounted to only 37% among non-users in the omnivorous group. Vitamin C is present in a wide range of food; hence, vitamin C deficiency should be rare. However, forty percent of participants did not reach the vitamin C RV with food alone. Insufficient intake of vitamin C was present in all groups, including vegans, where intakes were the highest and which were also reported to have the highest intakes of vitamin C by the literature [7]. Low intake of fruit and vegetables is generally observed in the Slovenian population [43]; additionally, high carbohydrate fruits and vegetables are intentionally excluded in the LCHF diet, and this is a risk factor for insufficient vitamin C intake with food. A recent representative study of the USA population showed a 22% decline in vitamin C intake across a 20-year period, mostly due to lower intake of fruit juices, while relatively low intakes of vegetables persisted [44]. As fruit juice contributes substantially to energy intake, its use was low in our sample and the preferable beverages were water and herbal tea.

Single-mineral DS use was rarely reported by our participants, except for DSs with magnesium. Insufficient magnesium intake with food alone was observed in 40% of participants. The lowest magnesium intake was in the LCHF group where only 40% of participants reached the RV with food alone, which is consistent with previous studies [8]. However, LCHF was also the group with the highest prevalence of magnesium DS use (50%). Magnesium supplementation in this group increased magnesium intake adequacy, but only in participants who took a magnesium DS and not a MMN DS, since the doses in the latter were too low (Figure 2C). Vegans, followed by vegetarians, had higher magnesium intake adequacy with food alone (81% and 65%, respectively), which is in line with previous outcomes [7].

Iron intake with food alone was the highest in the vegan group, where 97% of participants reached the RV, followed by the omnivorous, LCHF, and vegetarian group (92%, 88% and 84%, respectively); however, the iron intake was not corrected for lower bioavailability from plant sources. The observed higher iron intakes of vegans, but not vegetarians, are in line with previous studies [7]. Reports also show that even though bioavailability of iron is higher from animal products, such as meat and meat products, and vegan diet contains more inhibitors of absorption, participants on the vegetarian or vegan diet had normal iron status due to high iron intake [45]. In contrast, iron intake in our vegetarian group was more comparable with the omnivorous and LCHF group, than with the vegan group (Table 2, Table 3, Table 4, Table 5, Table 6, Table 7, Table 8, Table 9 and Table 10). Higher iron intake in vegans might show better awareness of low iron bioavailability in their diet, which is also shown in the case of vitamin B_12_ supplementation and points to the need of raising awareness in the vegetarian group.

### 4.2. Inadequate Intake with Food and Low or No Supplementation

Intake adequacy for some micronutrients was low, and many of them were rarely supplemented, and even when they were, the DS had insufficient doses.

Folate intake with food sources alone was insufficient in more than 40% of our participants. The Slovenian representative sample reports an even higher prevalence of folate insufficiency, with 58% [43]. The vegan group had the highest folate intake, with 81% of participants reaching RV, followed by vegetarians, omnivores, and those practicing LCHF. Contrary to Swedish reports [8], our LCHF participants had low folate intakes with food alone, with 58% of participants not reaching the RV. Only 22% of participants used folate DS. Importantly, everyone who took DSs with folate reached the RV, regardless of the type of DS: single-vitamin DS or MMN DS with folate. This shows that in case folate DSs were used, they would be properly chosen and dosed; however, almost half of participants who needed folate supplementation did not take it.

Pantothenic acid intake was low in our participants; only 31% reached the RV and low supplementation was also seen. The highest intake of pantothenic acid with food alone was observed in the LCHF group with half of participants with adequate intake. Interestingly, the LCHF group also most commonly used DSs with pantothenic acid. In total, 20% of all participants supplemented pantothenic acid, the majority of them as part of a MMN DS. Almost all participants (92%) who took a DS with pantothenic acid reached the RV. With this in mind, foods high in pantothenic acid and/or suitable DSs should be promoted more.

DSs with potassium are rare in Slovenia and so is potassium DS use; only three (2.3%) participants supplemented potassium, two of them as part of a MMN DS. Less than a quarter of participants reached the RV for potassium with food alone and no one who took potassium DSs increased potassium intake enough to reach the RV. The latest data showed that potassium intake is low in Europe [46]. Potassium DSs in Slovenia are rare and have low doses in comparison to the Slovene recommended intake, the highest dose we found was 375 mg, which is only 19% of the recommended daily intake, despite reports that potassium supplementation up to 3 g per day showed no adverse effects [25].

The prevalence of calcium supplementation with DS was low in our sample (10%), despite low calcium intake with food alone in all dietary groups; only 24% of participants reached the RV. The highest prevalence of adequate calcium intake was in the omnivorous group, 43% met RV, followed by LCHF, vegetarian, and vegan (25%, 24%, and 13%, respectively). Low calcium intake was previously reported among vegetarians and was even lower among vegans [7], but in our study we also observed low calcium intake in the LCHF and omnivorous group, which is in contrast to previous studies reporting significantly lower calcium intake in vegans compared with vegetarians and omnivores [7,41]. It is concerning that among calcium DS users, 31% of participants did not reach the RV despite calcium supplementation. Low calcium supplementation points to a low awareness of low calcium intake in all dietary groups, because calcium DSs are widely available in different doses: 60%, 80%, and 100% of the RV.

Molybdenum intake in our participants was relatively low, only 30% of participants reached the RV. Molybdenum was also rarely supplemented, only 8% of participants supplemented it, all with a MMN DS. MMN DSs with molybdenum had low doses and only 50% of participants who supplemented molybdenum reached the RV.

Only ten participants of our study reached the iodine RV, only two of them without a DS. Iodine is systematically supplemented in Slovenia through salt iodization; therefore, iodine is rarely present in DSs. Iodine sufficiency is periodically assessed in a representative sample of the population, for which sufficient iodine status is reported due to highly excessive salt intake [47]. Our participants had lower salt intake than reported for the general population in Slovenia [48]. Low iodine intake was reported before along with low urinary excretion of iodine in vegans but also in the vegetarian and omnivorous group [32,41]. Iodine intakes in our participants could be even lower than assessed, as non-iodized salt is on the market and some laic nutritional information sources preferred by people on special dietary patterns, especially vegetarians and vegans, promote it as more natural and healthy [49]. Eleven participants supplemented iodine as part of a MMN DS.

### 4.3. Multimicronutrient Dietary Supplements (MMN DS)

MMN DSs were very commonly used, more than a quarter of participants took them, as was also reported before in [50,51]. The LCHF group had the highest prevalence of MMN DS use among groups (54%), followed by the vegan and vegetarian groups (28% and 27%, respectively) and the lowest prevalence of MMN DS use was in the omnivorous group (8%). Three MMN DSs were used by more than one participant: the first MMN DS included both vitamins and minerals, the second MMN DS included only vitamins from group B, and the third MMN DS was composed of vitamin C, thiamin, riboflavin, and niacin (Figure 2A).

MMN DSs had a wide range of included vitamins and minerals and a wide range of dosages. Of the micronutrients that we identified to have the lowest prevalence of adequate intake with food, vitamin D was found in only ten MMN DSs used by our participants, only two of which contained an adequate daily amount of vitamin D. It was previously reported that vitamin D is often present in a low dose in MMN DSs, nevertheless the incidence of deficiency in vitamin D was also decreased with MMN DS use [51]. Calcium was found in ten MMN DSs and the highest amount of calcium in a MMN DS was 20% of the RV. Low calcium intake adequacy and also low calcium intake with a MMN DS was reported previously [50,51], which indicates the need to use a calcium-only DS to reach sufficient intakes. Furthermore, potassium was only present in two MMN DSs with amounts of only 1 and 2% of the RV, which was also indicated by other authors [51]. We observed that some micronutrients were supplemented only in the form of MMN DSs, these were potassium, calcium, chromium, molybdenum, iodine, and vitamins from group B, except vitamin B_12_.

Some micronutrients are often present in MMN DSs and also in an adequate amount, such as vitamins from group B and vitamin E. MMN DSs often improved adequacy of micronutrient intake of vitamins and minerals. In our study, this was mostly seen for group B vitamins. B group vitamins were also those that most commonly exceeded the UL. DS use increased the prevalence of exceeded UL for niacin, folate, and pyridoxine, which is in line with previous reports [19,52]. On the other hand, MMN DSs were often missing vitamins and minerals that were shown to have low adequacy in many dietary groups, such as vitamin D, potassium, calcium, and iodine.

### 4.4. Adequacy of Supplementation

DS use was common, but often did not answer the needs of an individual or dietary pattern. Some micronutrients were correctly chosen and supplemented, such as vitamin B_12_ and vitamin D. The use of DSs with vitamin B_12_ and vitamin D increased the prevalence of adequate intake of those vitamins, even if doses were often misaligned with the need or the RV. Vitamin D was insufficiently (with too low a dose) supplemented in 32% of vitamin D DS users through the whole year and also in 23% in the winter months. On the other hand, vitamin B_12_ was often supplemented in extra high doses, which is not recommended, despite the absence of a UL for vitamin B_12_. Furthermore, some micronutrients were supplemented despite adequate intake with food. For example, among vitamin K DS users, 87% of them had adequate intake with food alone. Use of MMN DSs with B group vitamins was frequent among the LCHF group, despite high intakes of riboflavin, biotin, and vitamin B_12_ with food alone. Riboflavin and biotin intakes were also adequate with food alone in omnivorous DS users. Iron intake with food alone was adequate in all iron DS users, except in DS users from the vegetarian group. On the contrary, we saw low supplementation of micronutrients that were not sufficiently provided with food, such as potassium and iodine. Low adequate intake with food alone and low supplementation were also observed for calcium in vegans, vegetarians, and the LCHF group, and for vitamin A and vitamin E in the omnivorous and vegetarian groups.

DS users were more likely to exceed the UL for niacin, zinc, folate, iron, pyridoxine, and vitamins D and C than nonusers. Exceeding the UL for niacin with DS use was previously reported [19], but the same study also reported an exceeded UL for folate, vitamin A, and iron with DS use, which in our study was present in very few cases. Exceeded UL in DS users for zinc was also previously reported [30]. Overall, exceeding the UL in our participants was lower than previously reported [51,52].

Our sample was carefully chosen to be able to compare groups, because previous vegan and vegetarian samples were associated with better lifestyle choices and this made those two groups hard to compare with others, especially a representative sample of omnivores. In the present study, we invited participants with an interest in nutrition, and our groups were matched for BMI, physical activity, fat, and fat-free mass, as well as smoking status. Furthermore, we also did not find any differences between DS users and nonusers in education level, income, gender, age, and diet quality, which also made the two groups easy to compare.

The present study pointed to many micronutrient intake shortages compared to reference values in different dietary patterns, but further research on the serum levels of micronutrients is needed. Dietary characteristics of different dietary patterns might influence bioavailability of micronutrients. Magnesium bioavailability, for example, is lower with high phytic acid intake, as often seen in the vegan group [53]. Iron bioavailability is higher from animal sources in the form of heme-iron, but it is also dependent on other dietary factors such as intake of vitamin C [54]. Additionally, some micronutrients in doses lower than the RV do not cause clinical effects; for example, molybdenum was researched for its positive effects on treating anemia and arthritis, but healthy adults are unlikely to develop clinical deficiencies [55].

## 5. Conclusions

We can conclude that DS use can increase the prevalence of micronutrient intake adequacy if DSs are chosen properly, which was not always the case. Vitamin B_12_ and vitamin D were supplemented to fill nutritional gaps, but the doses were not always appropriate. The fact that several nutrients, such as vitamin K, riboflavin, biotin, and iron were supplemented needlessly, whereas other nutrients, such as potassium, calcium, and iodine were not supplemented, even if intakes with food alone were insufficient, indicates a need for more accurate public education. While it is obvious that participants following diets that omit whole food groups are well aware of certain shortages, such as vitamin B_12_ and iron in the vegan group, and, to a degree, vitamin C and calcium in the LCHF group, the other risks of inadequate intakes are not well known. Increased awareness of possible diet shortages and how to fill them is needed among people without dietary supervision, where it should be acknowledged that several dietary components, typical for the investigated dietary patterns, may influence the micronutrient bioavailability.

Regardless of diet, inadequate micronutrient intake is common among participants who hold the opinion that their diet is balanced and beneficial to health. This raises the need for food counseling and help with selecting proper DSs with a suitable dose. Also, certain adjustments of MMN DSs’ composition, such as decreased doses of B group vitamins and increased potassium and vitamin D doses, could be considered.

## Figures and Tables

**Table 1:** Study participants’ characteristics.

Variable (Unit)	All	Omnivorous	Vegan	Vegetarian	LCHF
N (Female)	130 (97)	37 (25)	32 (23)	37 (30)	24 (19)
Age (years) *	37 ± 10	36 ± 12 ^c^	34 ± 10 ^e^	37 ± 11 ^f^	41 ± 6
Body mass (kg)	65 ± 11	66 ± 13	63 ± 8	65 ± 10	68 ± 13
BMI (kg/m^2^)	22.5 ± 2.8	22.6 ± 3.0	21.8 ± 2.1	22.4 ± 2.6	23.4 ± 3.1
Nutritional intakes					
Energy (kcal)	2060 ± 630	2160 ± 670	2070 ± 650	1960 ± 630	2030 ± 530
Protein (g) *	80 ± 37	91 ± 42 ^a,b,c^	65 ± 31 ^e^	65 ± 23 ^f^	108 ± 35
CHO (g) *	230 ± 130	250 ± 110 ^a,c^	310 ± 120 ^d,e^	250 ± 110 ^f^	51 ± 35
Fat (g) *	88 ± 42	81 ± 20 ^a,c^	64 ± 27 ^d,e^	76 ± 27 ^f^	151 ± 46
Fibers (g) *	32 ± 28	27 ± 15 ^a,c^	50 ± 43 ^d,e^	32 ± 15 ^f^	18 ± 21

LCHF—low-carbohydrate high-fat; N—numerus; BMI—body mass index; CHO—carbohydrate. All data are provided as means ± standard deviation except for the numerus. *—*p* < 0.05, Kruskal–Wallis H test; ^a–f^—*p* < 0.05, Mann–Whitney test between ^a^—omnivorous and vegan group; ^b^—omnivorous and vegetarian group; ^c^—omnivorous and LCHF group; ^d^—vegan and vegetarian group; ^e^—vegan and LCHF group; ^f^—vegetarian and LCHF group.

## Data Availability

The raw data supporting the conclusions of this article will be made available by the authors on request.
